# A Dual Band Frequency Reconfigurable Origami Magic Cube Antenna for Wireless Sensor Network Applications

**DOI:** 10.3390/s17112675

**Published:** 2017-11-20

**Authors:** Syed Imran Hussain Shah, Sungjoon Lim

**Affiliations:** School of Electrical and Electronics Engineering, College of Engineering, Chung-Ang University, 84 Heukseok-ro, Dongjak-gu, Seoul 156-756, Korea; engr.shahsyedimran@gmail.com

**Keywords:** frequency switchable antenna, origami magic cube, meandered monopole, wireless sensor networks

## Abstract

In this paper, a novel dual band frequency reconfigurable antenna using an origami magic cube is proposed for wireless sensor network (WSN) applications. The proposed origami antenna consists of a meandered monopole folded onto three sides of the magic cube. A microstrip open-ended stub is loaded on the meandered monopole. The proposed origami magic cube can be mechanically folded and unfolded. The proposed antenna operates at 1.57 GHZ and 2.4 GHz in the folded state. In the unfolded state, the proposed antenna operates at 900 MHz and 2.3 GHz. The resonant frequency of the second band can be tunable by varying the length and position of the open stub. The origami magic cube is built on paper. Its performance is numerically and experimentally demonstrated from S-parameters and radiation patterns. The measured 10 dB impedance bandwidth of the proposed origami antenna is 18% (900–1120 MHz) and 15% (2.1–2.45 GHz) for the unfolded state and 20% (1.3–1.6 GHz) and 14% (2.3–2.5 GHz) for the folded state. The measured peak gain at 900 MHz and 2.3 GHz are 1.1 dBi and 2.32 dBi, respectively, in the unfolded state. The measured peak gain at 1.5 GHz and 2.4 GHz are 3.28 dBi and 1.98 dBi, respectively, in the folded state.

## 1. Introduction 

During the last few decades, wireless communication has shown a tremendous growth. Many wireless applications have been integrated into a single radio device. For such radio devices, multiple antennas for various frequencies are not a suitable option because they would require a large amount of space with sufficient distance between the multiple antennas to avoid interference. Therefore, the antennas used in these radio devices should be multiband, wideband, or frequency reconfigurable. Compared to multiband and wideband antennas, a frequency reconfigurable antenna is an attractive choice because it can minimize the interference between multiple frequencies [1]. The monopole antennas have received great attention from last few decades because of its interesting features like omnidirectional radiation characteristics, compact size (λ/4), low cost, easy fabrication procedure and easy integration with other microwave circuits. Planar monopole antennas are mostly fed by microstrip line or coplanar waveguide. Due to its image in the ground plane, radiation pattern of the monopole antenna is similar to the dipole antenna. Dual band and multiband monopole antennas can play an important role in modern multiband communication systems. In [2], a compact monopole patch antenna with very interesting feature of wideband impedance bandwidth is presented. In [3], switching techniques for notched ultra-wide band monopole antenna is presented. In [4,5], a 3-dimensional (3-D) cubic antenna is presented for radio frequency identification (RFID) and wireless sensor networks (WSN). The antenna was fabricated on six planar sides of cube. The cube was realized by using flexible liquid crystal polymer (LCP) and paper which serve as substrate part of the antenna.

Origami is a Japanese word composed of two terms Ori and Kami. Ori means folding and Kami means paper. Therefore, the word origami is used for paper folding [6]. It is an ancient art and has been used for various applications during the last few years [7]. Origami is attracting significant attention from researchers owing to its several advantages including deployability, easy fabrication procedure (folding), easy miniaturization, and a low fabrication cost. Recently, origami has been used for architecture and space-born applications as well as in various mechanical engineering designs [8,9]. The identified origami features are advantageous for antenna design applications where portability and deployability of the antenna is needed. Advancement in various simulation tools enables analysis and design techniques using the origami concept for antenna applications. Origami folding in one dimension, two dimensions, and three dimensions can be used to efficiently design an antenna that would not be possible by conventional antenna fabrication procedures [10,11]. A literature review reveals a few antennas that use the origami concept. For instance, in [12], a high-gain antenna was presented by using an origami tetrahedron structure. In [13], a deployable origami yagi antenna was presented but the antenna could be unfolded only by using a servo motor. A self-folding microstrip patch antenna was presented in [14]. A circularly polarized origami antenna was proposed for military field deployment [15]. In [16,17], a frequency reconfigurable origami antenna was presented.

In this paper, a novel dual band frequency reconfigurable monopole antenna using an origami magic cube is proposed. The proposed origami antenna consists of an open-ended stub loaded meandered monopole designed on the magic cube. The proposed origami magic cube antenna can be folded and unfolded. The resonant frequencies for both bands are reconfigurable by folding and unfolding the magic cube. Therefore, the proposed antenna operates at 1.57 GHz and 2.4 GHz in the folded state. In the unfolded state, the proposed antenna operates at 900 MHz and 2.3 GHz. The measured 10 dB impedance bandwidth of the proposed origami antenna is 18% (900–1120 MHz) and 15% (2.1–2.45 GHz) for the unfolded state and 20% (1.3–1.6 GHz) and 14% (2.3–2.5 GHz) for the folded state. The measured peak gain at 900 MHz and 2.3 GHz are 1.1 dBi and 2.32 dBi, respectively, in the unfolded state. The measured peak gain at 1.57 GHz and 2.4 GHz are 3.28 dBi and 1.98 dBi, respectively, in the folded state. The origami magic cube is built on paper and, therefore, this is a low-cost antenna. The fabrication procedure for the antenna is very easy and fast. For outdoor WSN applications, the antenna must be stable and robust. Compared to the previous origami antennas such as [16,17], the proposed antenna is stable and robust. For instance, the origami antennas in [16,17] were made by folding a single sheet of paper. Thus, they are not very stable after repeated folding and unfolding. On the other hand, the proposed antenna is made by folding two paper sheets together and the magic cube geometry makes it robust in both the folded and unfolded state. The 3-D cubic shape is very suitable for WSN. The cubic shape allows for smart packing to easily integrate the sensor equipment [4]. Secondly, the proposed antenna provides quasi isotropic radiation pattern. Therefore, these two features (3-D cubic shape and its quasi isotropic radiation pattern) makes it suitable for wireless sensor networks. For example, the proposed antenna can be used in WSN for real time tracking and monitoring the environmental conditions. The proposed antenna can be instantly designed even without any EM simulator, by folding the two paper sheets in a magic cube shape and realizing a monopole of length (λ/4) for the desired frequency of operation. Therefore, it is very suitable for amateur radio for experimental purposes. On the other hand, the Internet-of-Things (IoT) is an emerging technology and it is estimated that 30 billion devices will be connected in 2020. Therefore, the low cost features and quasi isotropic radiation pattern of the proposed antenna make it a potential candidate for IoT applications.

## 2. Origami Magic Cube Antenna Design and Fabrication

**Figure 1:**
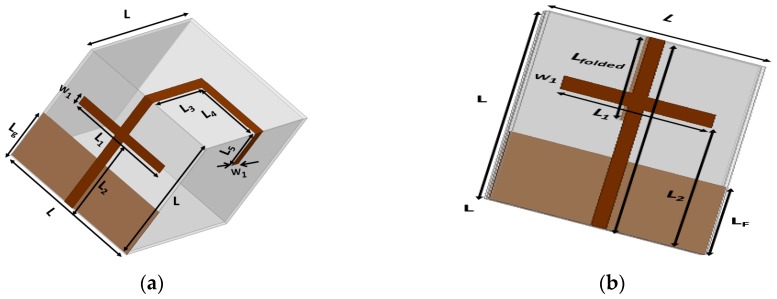
Proposed origami magic cube antenna (**a**) in the unfolded state (**b**) magic cube antenna in the folded state (L = 50 mm, L_1_ = 24 mm, L_g_ = 20 mm, L_2_ = 30 mm, L_3_ = L_4_ = 25 mm, L_5_ = 15 mm, W_1_ = 2 mm, and L_folded_ = 25 mm).

**Figure 2:**
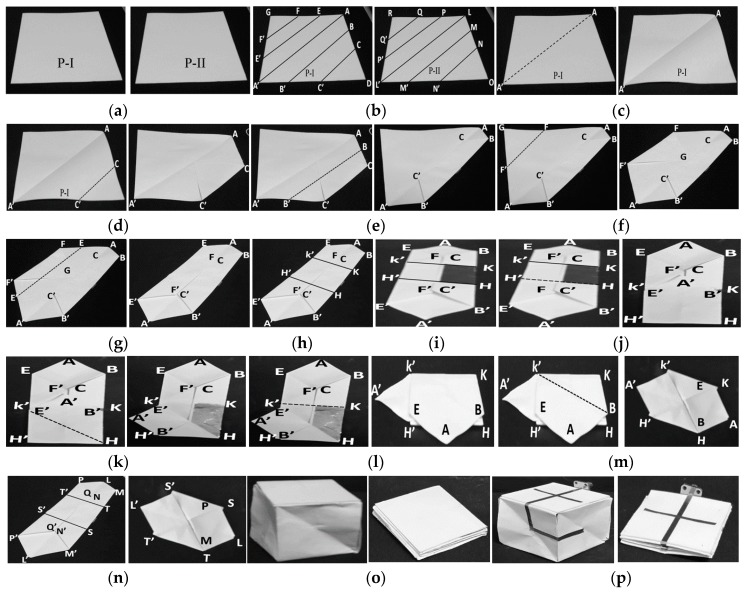
Antenna fabrication steps: (**a**) selection of two paper sheets; (**b**) sheets marked with alphabets; (**c**) folding sheet P-I along AA’; (**d**) folding along CC’; (**e**) folding along BB’; (**f**) folding along FF’; (**g**) folding along EE’; (**h**) dividing paper in three sections; (**i**) realization of the ground plane; (**j**) folding along H’H; (**k**) folding along K’H; (**l**) folding along KK’; (**m**) folding along K’B; (**n**) folded paper sheet P-II; (**o**) completed origami magic cube in unfolded and folded states; (**p**) final antenna prototype in unfolded and folded states.

The presented antenna is designed on a paper substrate in an origami magic cube shape. The geometry of the proposed antenna in the folded and unfolded forms is presented in Figure 1. First, the origami magic cube is built and then the monopole is realized on the magic cube. The design procedure for the magic cube can be divided into several steps as shown in Figure 2. First, two different square paper sheets with the same area (140 mm × 140 mm) and a thickness of 0.2 mm are selected. These sheets are labeled P-I and P-II. Sheet P-I is labeled with the segments AA′, BB′, CC′, DD′, FF′, G and D. Sheet P-II is marked with segments LL′, MM′, NN′, PP′, QQ′, R and O as shown in Figure 2b. Sheet P-I is folded and unfolded across/along AA’.

This creates a crease which divides the paper into two triangular sections as shown in Figure 2c. Then, the paper is folded step by step along CC’, BB’, FF’, EE’ as shown in Figure 2d–g. These folding steps convert the paper into the hexagonal shape shown in Figure 2g. The hexagonal shaped paper consists of two longer sides BB’ and EE’ and four smaller sides AB, AE, A’B’, and A’E’. Next, the hexagonal shaped paper is divided into three sections. These sections consist of an upper section KK’EAB, a middle section HH’KK’, and a lower section HH’E’A’B’ as shown in Figure 2h. A copper strip with dimensions of L_g_ × L is attached to the middle section (HH’KK’) of the hexagonal shaped paper to serve as the ground plane in the proposed antenna as shown in Figure 2i. In Figure 2j, the lower section of the hexagonal shaped paper, labeled as HH’E’A’B, is folded towards the middle section of the paper, labeled as HH’KK’. This folding converts the hexagonal shaped paper into a pentagonal shape. After this, section A’B’HE’ is folded towards HH’K’ and section K’KBAE is folded towards HH’KK’ as shown in Figure 2k–l. Next, section BAEK’ is folded towards section BKK’ to convert it into a hexagonal shape as shown in Figure 2m. This hexagonal shaped paper has three sections. The bottom section consists of a square KK’HH’ and the top consists of two tetrahedron sections K’KAH and K’A’H’H. Then sheet P-II is chosen which has been labeled with sections LL′, MM′, NN′, PP′, QQ′, R, and O as shown in Figure 2b. The folding steps mentioned for sheet P-I are repeated for sheet P-II to get another hexagonal shaped folded paper. The final folded shape of sheet P-II is shown in Figure 2n. In the next step, the folded sheets P-I and P-II are joined together to form a magic cube. The magic cube has six square-shaped sides. The dimensions of all sides are the same. This magic cube can be folded and unfolded. The magic cube in the unfolded and folded states is shown in Figure 2o. After building a magic cube, the monopole is implemented on three sides of the magic cube. Copper film having conductivity 4.4 × 105 S/m and a thickness of 0.1 mm is used for realization of the monopole. A monopole antenna is realized by copper film because it does not crack for repeating folding and unfolding process. The final antenna prototype in unfolded and folded states are shown in Figure 2p. The ANSYS High Frequency Structure Simulator (HFSS) is used for electromagnetic simulation and analysis of the proposed dual band frequency switchable antenna. We used paper as a substrate in our magic cube antenna fabrication. Firstly, we characterized the paper by designing a T-resonator for our desired frequency range. In the desired frequency range (0 to 3 GHz), the dielectric constant and dielectric loss tangent of the paper was 2.2 and 0.04, respectively.

**Figure 3:**
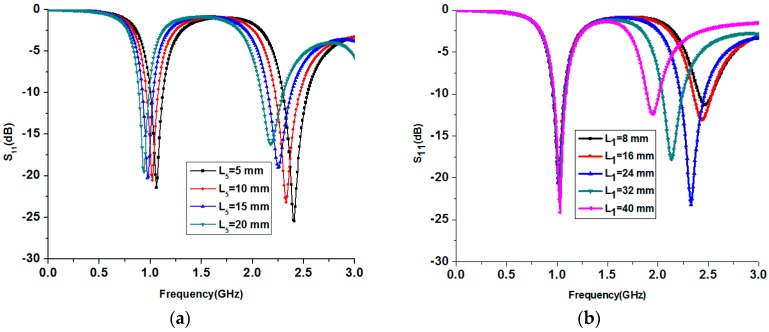

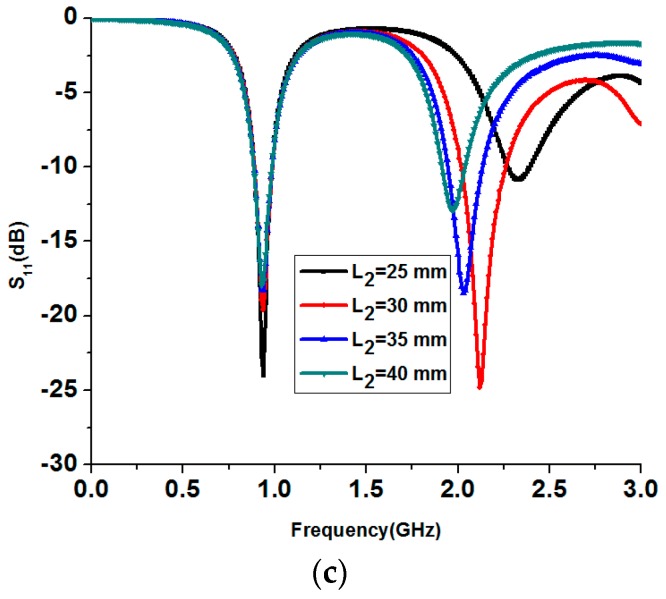
(**a**) Parametric study for L_5_ in the unfolded state; (**b**) parametric study for length of the stub (L_1_); (**c**) parametric study for position of stub (L_2_).

A parametric study is performed to determine the length of the monopole arm L_5_ required to get the desired resonant frequency of 900 MHz in the unfolded state as presented in Figure 3a. When L_5_ is increased, the resonant frequency of the monopole decreases. In this study, L_5_ is chosen as 15 mm to get the desired operation frequency of 900 MHz in the unfolded state. The parametric study for various lengths (L_1_) of the open stub of the monopole is presented in Figure 3b. As can be seen from the S-parameters of the various lengths (L_1_) of the open stub, the resonant frequency of the second band can be easily controlled by varying the length of the open stub. In this study, L_1_ is chosen as 24 mm to get an operation frequency around 2400 MHz.

The position of the open-ended stub can also be varied to select the desired operation frequency as shown in Figure 3c. Based on this study, L_2_ is chosen as 30 mm in order to operate the antenna around 2400 MHz.

## 3. Measurement Results

**Figure 4:**
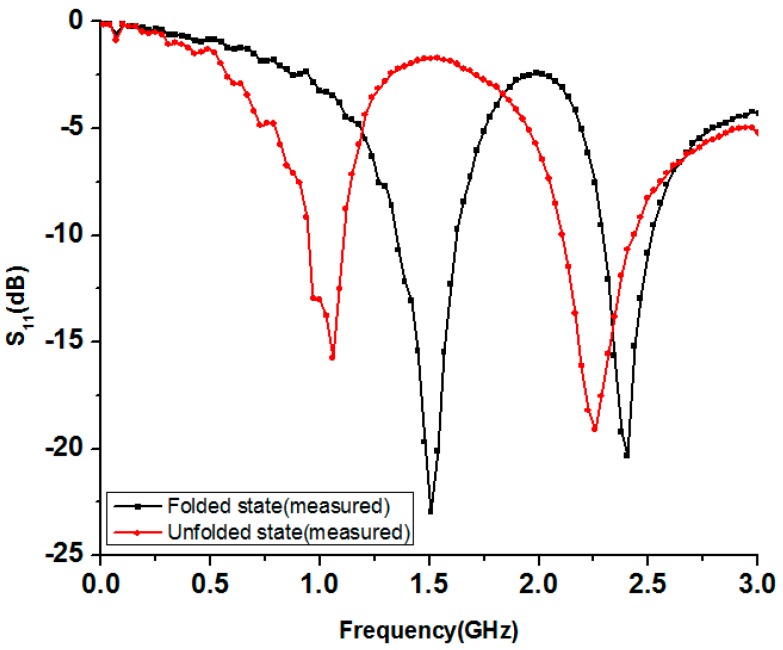
Measured reflection coefficients of the proposed origami magic cube antenna in the folded and unfolded states.

After the parametric study, the proposed antenna is fabricated with the optimal dimensions for the required resonant frequencies. The antenna prototype is built on the origami magic cube and displayed in Figure 2p in the unfolded and folded states. The reflection coefficients of the antenna for the folded and unfolded states are measured using an MS2038C vector network analyzer (Anritsu, Cary, NC, USA). The simulated and measured reflection coefficients of the proposed origami magic cube antenna are presented in Figure 4 for the folded and unfolded states. The proposed antenna operates at 1.57 GHz and 2.4 GHz in the folded state. In the unfolded state, the proposed antenna operates at 900 MHz and 2.3 GHz. The measured 10 dB impedance bandwidth of the proposed origami antenna is 18% (900–1120 MHz) and 15% (2.1–2.45 GHz) for the unfolded state and 20% (1.3–1.6 GHz) and 14% (2.3–2.5 GHz) for the folded state.

**Figure 5:**
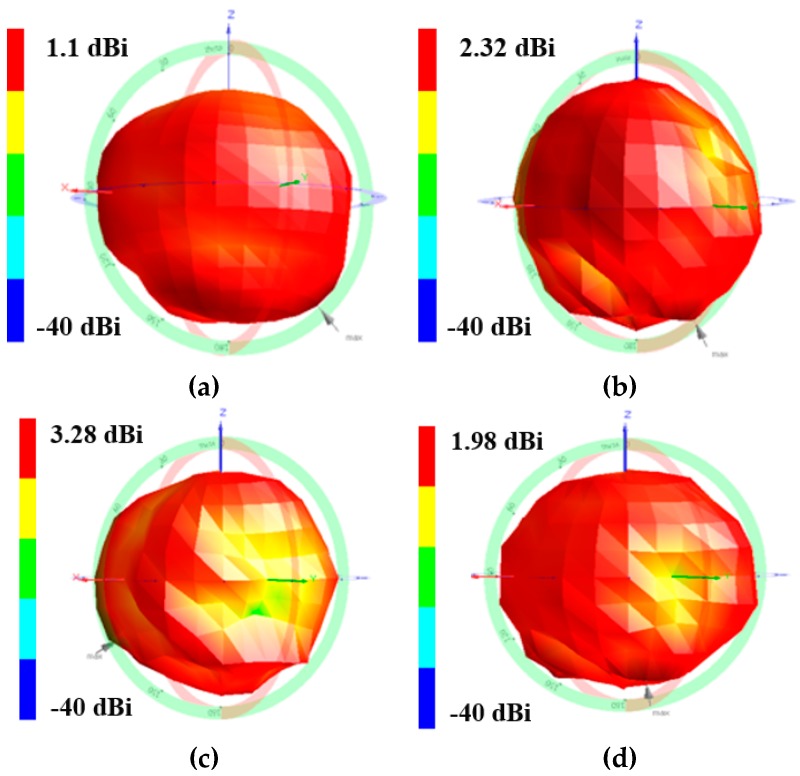
Measured 3D radiation patterns of the proposed origami magic cube antenna in the unfolded state (**a**,**b**): (**a**) at 900 MHz, (**b**) 2200 MHz; measured 3D radiation patterns of the proposed origami magic cube antenna in the folded state (**c**,**d**): (**c**) 1500 MHz, (**d**) 2400 MHz.

The radiation pattern of the antenna is measured in an anechoic chamber for all the operational frequencies in the folded and unfolded states. In Figure 5, the measured 3D radiation pattern of the antenna is presented for the frequencies of 900 MHz and 2200 MHz in the unfolded state. The antenna shows a measured peak gain of 1.1 dBi and 2.32 dBi at 900 MHz and 2200 MHz, respectively, in the unfolded state. Next, the proposed antenna is folded and the 3D radiation pattern of the antenna is measured in the folded state for the frequencies of 1.5 GHz and 2.4 GHz. The antenna shows a peak gain of 3.28 dBi and 1.98 dBi for the frequencies of 1.5 GHz and 2.4 GHz, respectively, in the folded state.

**Figure 6:**
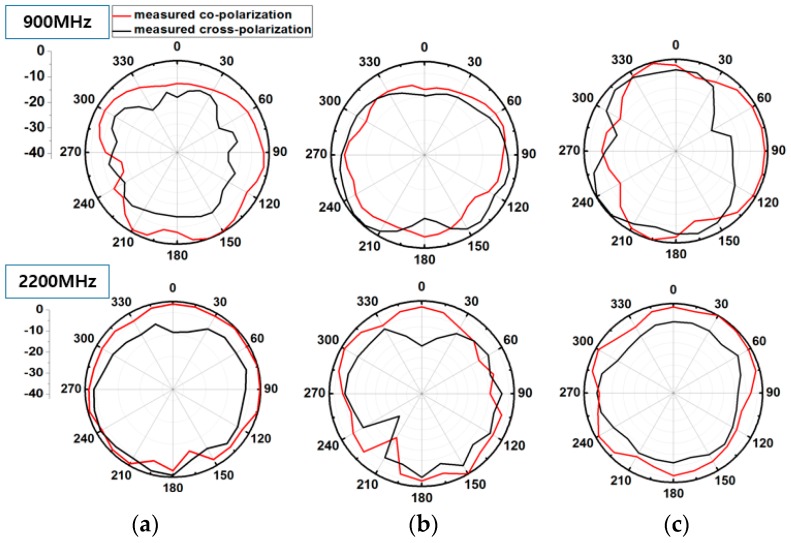
Measured 2D normalized radiation patterns of the proposed origami magic cube antenna in the unfolded state. (**a**) yz-plane, (**b**) xz-plane, (**c**) xy-plane.

**Figure 7:**
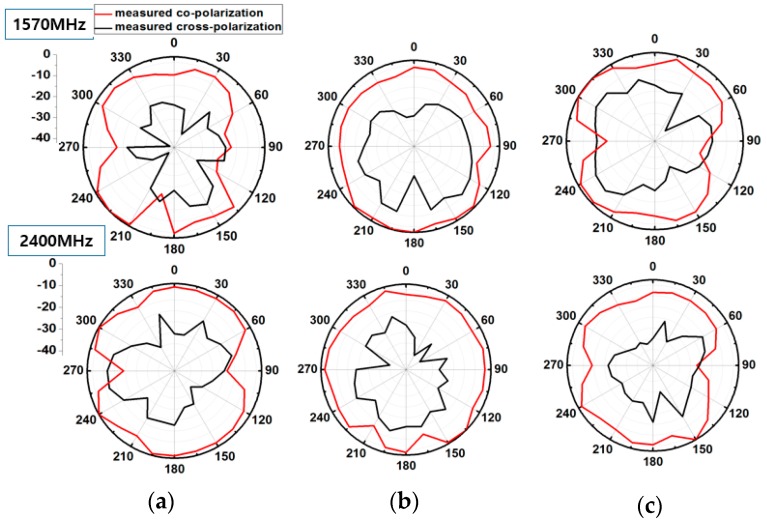
Measured 2D normalized radiation patterns of the proposed origami magic cube antenna in the folded state. (**a**) yz-plane, (**b**) xz-plane, (**c**) xy plane.

The measured radiation pattern provides quasi isotropic radiation pattern especially in the unfolded state where gain deviation (difference between maximum and minimum of gain) is even less than 14 dBi. This feature makes it suitable for WSN applications. Gain deviation in the unfolded state is smaller because a meandered monopole which is folded onto three sides of the magic cube fully contributes in radiation. In the folded state, some portion of meandered monopole is overlapped resulting in reduced electrical length. Therefore, in the folded state, gain deviation is more than the unfolded state. In Figure 6, the measured normalized radiation pattern of the antenna is shown for the frequencies of 900 MHz and 2200 MHz in the unfolded state. The measured normalized radiation pattern of the proposed antenna is plotted for the frequencies of 1500 MHz and 2400 MHz in the folded state in Figure 7. In the folded state, the height of the antenna is reduced by 90%.

## 4. Conclusions

A novel dual band frequency reconfigurable origami magic cube antenna was proposed. The proposed origami antenna consists of a stub loaded microstrip fed meandered monopole. The proposed origami magic cube antenna can be folded and unfolded to reconfigure the operating frequencies. The proposed antenna operates at 1.57 GHz and 2.4 GHz in the folded state. In the unfolded state, the proposed antenna operates at 900 MHz and 2.3 GHz. The origami magic cube is constructed out of paper and, therefore, this is a low-cost antenna. The fabrication procedure for the antenna is very easy and fast. The antenna is suitable for applications where portability and deployability of the antenna is required. Therefore, the proposed antenna is a good candidate for the internet of things (IoT), amateur radio, and WSN applications. In this work, we manually deformed the origami geometry without the actuator in order to demonstrate any feasibility. As a future work, we are going to integrate the proposed origami antenna with a pneumatic actuator.
